# Antioxidant activity of vine tea *(Ampelopsis grossedentata)* extract on lipid and protein oxidation in cooked mixed pork patties during refrigerated storage

**DOI:** 10.1002/fsn3.1013

**Published:** 2019-04-02

**Authors:** Xuan Zhang, Yu Xu, Hai Xue, Guo‐Chuan Jiang, Xue‐Jun Liu

**Affiliations:** ^1^ College of Food Science and Engineering Jilin Agricultural University Changchun China

**Keywords:** *Ampelopsis grossedentata*, antioxidant activity, cooked mixed pork patties, lipid and protein oxidation, vine tea extract

## Abstract

To prevent oxidation and maintain the quality of meat products, it is essential to add antioxidants. The aim of this work was to investigate the antioxidant activity of vine tea (*Ampelopsis grossedentata*) extract (VTE) and evaluate the effects of VTE on the quality characteristics and lipid and protein oxidation of cooked mixed pork patties during refrigerated storage. VTE had a significant DPPH radical scavenging activity, and its IC_50_ was 15.35 µg/ml. VTE‐treated mixed pork patties had a better texture than that of the control group (*p* < 0.05). VTE could significantly inhibit an increase in the TBARS value and the formation of carbonyl compounds (*p* < 0.05), and the inhibition was stronger than that of the butylated hydroxytoluene (BHT) group (*p* < 0.05), while the amount of sulfhydryl groups significantly decreased (*p* < 0.05). The color of VTE itself made the mixed pork patties darker (*p* < 0.05), but this did not affect the sensory scores and overall acceptability of the VTE‐treated patties, indicating the VTE can be incorporated into mixed pork patties. The scanning electron microscopy (*SEM*) results showed that the VTE inhibited the oxidation of the cooked mixed pork patties during refrigerated storage. These findings may be significant to helping extend the shelf life of meat products.

## 1 INTRODUCTION

Meat products are rich in nutrients (Biesalski, 2005), such as proteins, lipids, vitamins, and minerals (Choe et al., 2018). However, studies have shown that meat products are prone to oxidation during storage, resulting in food spoilage (Silva et al., 2018). The shelf life and quality of meat products are mainly affected by lipid and protein oxidation (Vuorela et al., 2005). Due to lipid oxidation, rancidity and off‐flavors will occur in meat products, which is due to the formation of the secondary oxidation end products (Lorenzo et al., 2018). The color, flavor, and texture of meat products also change (Gómez & Lorenzo, 2012). Results have shown that protein oxidation leads to an increase in carbonyl content and a decrease in sulfhydryl content (Bekhit, Hopkins, Fahri, & Ponnampalam, 2013). Therefore, antioxidants are usually added to meat products, which can inhibit the occurrence of oxidation and maintain product quality (Kumar, Yadav, Ahmad, & Narsaiah, 2015).

In recent years, synthetic antioxidants, such as butylated hydroxyanisole (BHA), butylated hydroxytoluene (BHT), and tertiary butyl hydroquinone (TBHQ), have been used in inhibiting meat oxidation (Fasseas, Mountzouris, Tarantilis, Polissiou, & Zervas, 2007). However, continuous use of synthetic antioxidants may increase carcinogenic potential (Hathway, 1966). Natural antioxidants have become a hot topic in recent years due to their health safety compared to that of synthetic antioxidants (Moyo, Oyedemi, Masika, & Muchenje, 2012). Vine tea (*Ampelopsis grossedentata*) is a plant belonging to Vitaceae and is widely distributed in southern China. Studies have shown that vine tea has good antioxidant activity (Gao et al., 2009). Vine tea extract (VTE) has a high scavenging activity toward DPPH free radicals (Gao et al., 2017). In addition, according to reports, VTE can significantly inhibit lipid oxidation in meat products (Ye, Wang, Duncan, Eigel, & O’Keefe, 2015).

Studies have shown that red meat is rich in nutrients and has many beneficial functions (Mafra et al., 2018). However, excessive intake of animal protein, especially red meat, can lead to increased intake of saturated fat and cholesterol, as well as excessive acid load (Mafra et al., 2018), which may increase the risk of disease, such as cardiovascular disease (Abete, Romaguera, Vieira, Lopez de Munain, & Norat, 2014), kidney disease (Mafra et al., 2018), type 2 diabetes (Pan et al., 2011), and cancer (Lippi, Mattiuzzi, & Cervellin, 2016), seriously threatening human health.

Soy protein meat analogues, as pure plant proteins, are generally obtained by special processing of protein concentrate and protein isolate (Palanisamy, Töpfl, Aganovic, & Berger, 2018); they have a fibrous structure and good mouthfeel (Krintiras, Göbel, Goot, & Stefanidis, 2015); and their amino acid composition ratio is close to that of the human body and thus can easily be absorbed and utilized. Because of their fiber texture and rich plant protein, it is necessary to add meat analogues in meat products. They can replace a portion of the animal proteins in human diets (Palanisamy et al., 2018), which not only meets the nutritional needs of people, but also reduces the intake of saturated fat and cholesterol (Kumar, Chatli, et al., 2015 2015), and reduces the risk of these diseases.

To compensate for the adverse effects of eating only red meat, mixed pork patties are made by adding meat analogues to pork patties. However, it has been found that mixed pork patties were prone to oxidation during refrigerated storage, which affected their quality and greatly limited their application.

This study investigated the antioxidant activity of VTE and the effects of VTE on the quality characteristics of mixed pork patties and the inhibition of VTE on lipid and protein oxidation in mixed pork patties during refrigerated storage. This study may be helpful in maintaining the quality and extending the shelf life of mixed pork patties.

## 2 MATERIALS AND METHODS

### 2.1 Materials

Vine tea was collected in May 2018 from Hunan Province of China (Northern latitude 29°66′, longitude 109°83′, altitude: 1,198 m). Soy protein meat analogues were purchased from Henan Pinzheng Food Technology Co., Ltd (Wen County, Jiaozuo, China), and they contained moisture (8.9%), protein (65.9%), and ash (4%) according to data from the manufacturer.

### 2.2 Preparation of vine tea extract (VTE)

Vine tea powders were extracted with petroleum ether and dried at 40°C to remove impurities, such as fats and pigments. The degreased powder was refluxed in 70% ethanol at 70°C for 1 hr. After filtering, the filtrate was concentrated to remove organic solvents and lyophilized to obtain vine tea extract (VTE).

### 2.3 Gas chromatography mass spectrometry (GC‐MS)

The flavonoids of VTE were determined by GC‐MS using a 6,890–5,975 gas chromatography mass spectrometer (Thermo Fisher Scientific Co., Ltd, Shanghai, China). Briefly, 10 mg of VTE was hydrolyzed with trifluoroacetic acid (TFA, 4 mol/L, 2 ml) at 110°C for 3 hr. The hydrolysate was dried under reduced pressure at 70°C. The hydrolyzed VTE was added to 1 ml of pyridine, and 1 ml of silylating reagent, N,O‐bis (trimethylsilyl) trifluoroacetamide (BSTFA) and trimethylchlorosilane (TMCS) (99:1), was added quickly. After oscillating to fully dissolve, the solution was reacted in a constant temperature oven at 50°C for 40 min, and then the supernatant was measured by GC‐MS.

The flavonoids of VTE were separated on the DB‐5MS column (30 m × 0.25 mm × 0.25 μm). The inlet temperature and interface temperature were 280°C, and column pressure was 73.0 κ. The column was set at a flow rate of 1 ml/min using helium as carrier gas, and the split ratio was 10:1. The column temperature was set as follows: the initial temperature was 80°C (for 3 min) and increased to 280°C at 10°C /min, then was held for 5 min, and the injection volume was 1 μl. The MS conditions were that the temperature of ion source was 200°C and the range of M/Z was 20–800.

### 2.4 Determination of total flavonoid content and DPPH radical scavenging activity of VTE

Total flavonoid contents were measured according to the method of Xie et al. (2015) with some modifications. Briefly, 0.1 ml solution of VTE was added to a 10 ml volumetric bottle, 3 ml of 5% AlCl_3_ solution was added, and volume was fixed with 95% ethanol. The absorbance of the sample was determined with a TU‐1901 ultraviolet‐visible spectrophotometer (Beijing Pu Analysis General Instrument Co., Ltd, Beijing, China) at 310 nm. The calibration curve was prepared by preparing dihydromyricetin solution with concentration of 5 to 25 μg/ml in 95% ethanol. The calibration curve was calculated as follows: *y* = 0.0571*x *− 0.0106, where *y* and *x* are the absorbance value and the concentration of the sample, respectively. The correlation coefficient (*R* ^2^) is 0.9947.

Two milliliter of various concentrations (20, 40, 100, 200, 400, and 800 μg/ml) of the sample solutions was thoroughly mixed with 2 ml of freshly prepared DPPH solution (0.1 mmol/L). The mixture was shaken vigorously and allowed to stand for 30 min in the dark, and the absorbance at 517 nm was then measured with a vis spectrophotometer (Pagenenal, Beijing, China). Ethanol was used as the blank control, and ascorbic acid was used as positive control. All tests were carried out in triplicate. The scavenging activity of VTE on DPPH is calculated according to the following formula:

(1) $$\text{DPPH radical scavenging activity(\textbackslash{}\% )}\;=\;1-\frac{A_{1}-A_{2}}{A_{0}}\times 100$$

where *A*_0_ is the absorbance of the incubation DPPH solution without addition of the sample or positive controls, *A*_1_ is the absorbance of the incubation mixture containing both the test sample and DPPH solution, and *A*_2_ is the absorbance of the sample without DPPH solution.

### 2.5 Preparation of mixed pork patties

Soy protein meat analogues were soaked with water until no hard cores remained. Excess water in the soy protein meat analogues was removed with a dehydrator, and then, the analogues were broken into pieces for use in the next step. 500 g of soy protein meat analogues and 500 g of pork back‐leg meat were used as the raw materials, and the formulation of mixed pork patties was as follows: 10% flour, 10% modified starch, 10% soy protein isolate, 3% salt, 2% white sugar, 2.5% monosodium glutamate, 0.3% pepper, and 7% water. Samples were homogenized in a chopper (Stephan, Shanghai, China) for 5 min after adding all the ingredients. The mixture was divided into four groups: (a) control group; (b) BHT group (0.01% BHT); (c) VTE 0.1 group (0.1% lyophilized VTE powders); and (d) VTE 0.3 group (0.3% lyophilized VTE powders). The four groups of mixtures were evenly mixed and formed into patties (length, width, and height were 10 cm, 5 cm, and 1 cm, respectively). The blended pork patties were baked for 20 min at 180°C and then at 210°C for 5 min in a universal oven (Rational, Beijing, China). The cooked samples were cooled and stored in oxygen‐permeable bags for 8 days at 4 ± 1°C. Indicators of the samples were determined after 0, 2, 4, 6, and 8 days of storage.

### 2.6 Analytical methods

#### 2.6.1 Proximate analysis

The proximate composition (moisture, protein, and fat contents) of the mixed pork patties was measured using a FOSS FoodScan™ Meat Analyzer Near‐Infrared Spectrophotometer (Model 78,810; Foss Co., Hillerød, Denmark) (Schillinga et al., 2018).

#### 2.6.2 The pH evaluation

Ten gram samples of mixed pork patties were homogenized with 90 ml of distilled water for 30 s using a homogenizer (IKA, Shanghai, China). Then, the pH of the mixed pork patties was evaluated with a pH meter (Mettler Toledo, Shanghai, China).

#### 2.6.3 Determination of texture characteristics

The texture characteristics of the samples were determined by a texture analyzer (FTC, Hangzhou, China). The same size samples (20 × 20 × 10 mm) were obtained from the mixed pork patties, and their texture characteristics, included hardness and springiness, were analyzed.

#### 2.6.4 Colorimetric determination

The colors of the mixed pork patties were measured using a color difference meter (HunterLab, Shanghai, China). The following color indices were measured: lightness (L^*^), redness (a^*^), and yellowness (b^*^). The chromatometer was calibrated with a white standard before use.

### 2.7 Lipid oxidation

#### 2.7.1 Thiobarbituric acid reactive substances (TBARS)

The TBARS value was evaluated based on the method of the official standard issued in China titled “Determination of malondialdehyde in food” (GB 2016‐5009.181).

### 2.8 Protein oxidation

Protein carbonyls were evaluated by the method of Oliver, Ahn, Moerman, Goldstein, and Stadtman (1987). Carbonyl content was determined by the reaction between 2,4‐dinitrophenylhydrazine (DNPH) and carbonyl group. Absorption was measured at 370 nm with a TU‐1901 ultraviolet‐visible spectrophotometer. The carbonyl content was calculated using the protein concentration and an extinction coefficient of 21.0 mM^−1^ cm^−1^, and the protein concentration was determined by using BSA as a standard, and the results were expressed as nmol of carbonyl/mg protein. The total sulfhydryl content was measured using 5,5′‐dithiobis (2‐nitrobenzoic acid) (DTNB) (Ellman, 1959). Absorption was measured at 420 nm with a TU‐1901 ultraviolet‐visible spectrophotometer. The results were calculated using the protein concentration at 280 nm and expressed as nmol sulfhydryl/mg protein.

### 2.9 Scanning electron microscopy

Scanning electron microscopy (*SEM*) was used to analyze the microstructure of all the groups. The *SEM* was evaluated by the method of Palanisamy et al. (2018). The refrigerated samples were taken out and cut into 10 × 10 mm, then dried immediately to the full. Then, the sample surfaces were sputter coated with gold and images were taken by XL‐30 ESEM FEG scanning electron microscope (FEI, Shanghai, China).

### 2.10 Sensory evaluation

Sensory evaluations such as flavor, color, mouthfeel, texture, and overall acceptance scores of the mixed pork patties stored for 0 days were evaluated. The assessment consisted of teachers and students (20–40 years old, 10 male and 10 female) from Jilin Agricultural University. The evaluation was conducted using a 5‐point scale (5 = great and 1 = bad).

### 2.11 Statistical analysis

All data are shown as the mean ± standard deviation (*SD*). The significant differences between groups were determined by the SPSS 24.0 system. The acceptable level for statistical significance was *p* < 0.05.

## 3 RESULTS AND DISCUSSION

### 3.1 Composition of Vine tea extract

**Figure 1:**
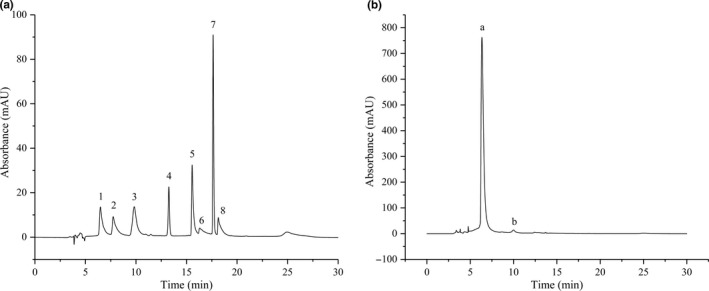
GC‐MS chromatograms of flavonoid standard compounds and VTE. (a) GC‐MS chromatograms of standards and (b) GC‐MS chromatograms of VTE. (1: dihydromyricetin; 2: rutin; 3: dihydroquercetin; 4: madendrin; 5: fructose; 6 quercetin; 7 apigenin; 8: kaempferol; a: dihydromyricetin; b: dihydroquercetin)

The GC‐MS chromatograms of VTE are shown in Figure 1a and b. According to the MS data, the flavonoid compounds were identified to be dihydromyricetin and dihydroquercetin. Dihydromyricetin was the main component of VTE, with an average content of 96.93%, while the average content of dihydroquercetin was 1.77%.

### 3.2 Total flavonoid content and DPPH radical scavenging activity of VTE

**Figure 2:**
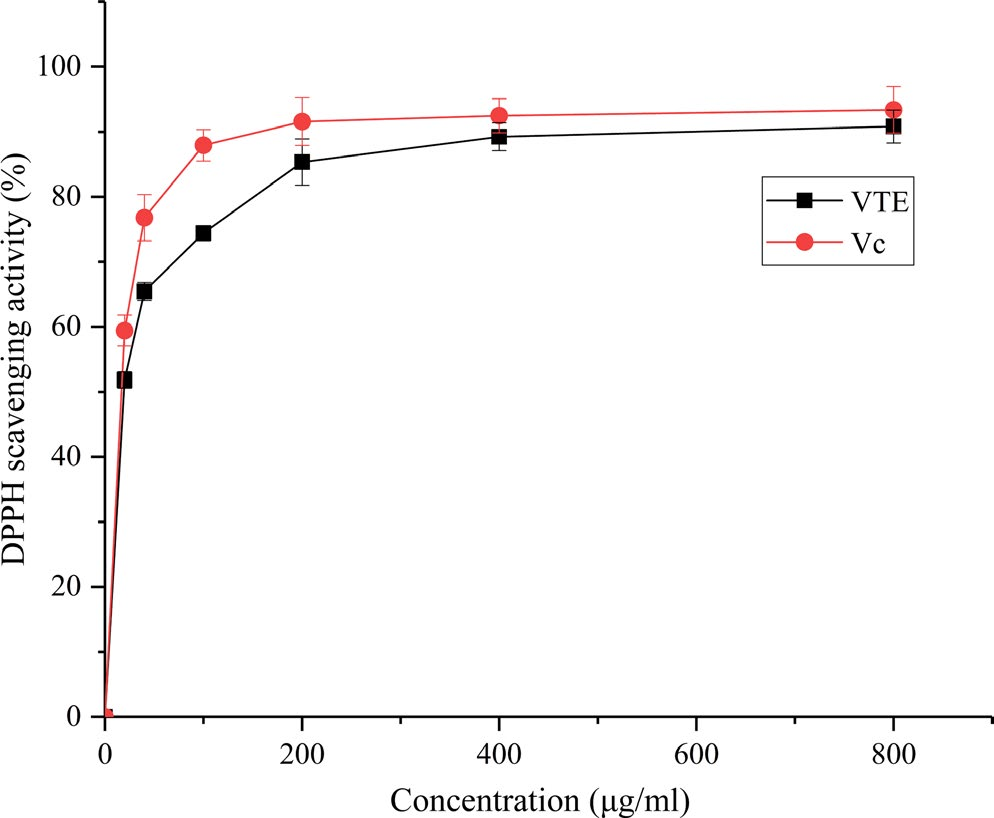
DPPH radical scavenging activity of VTE. All values are presented as the mean ± *SD* of three independent experiments

Using dihydromyricetin as the standard, VTE had a high flavonoid content of 8.14 g per 100 g of VTE. The DPPH radical is commonly used to estimate the antioxidant activity of antioxidants (Xie et al., 2015). The scavenging activity of VTE against DPPH radical is shown in Figure 2. VTE had a strong DPPH radical scavenging activity. When the concentration was 20 and 800 µg/ml, the scavenging rates were 51.84% and 90.82%, respectively (Figure 2). The IC_50_ for DPPH radical scavenging activity was 15.35 µg/ml. The DPPH radical scavenging activity of VTE increased in a concentration‐dependent manner.

### 3.3 Chemical composition of mixed pork patties

The protein, fat, and moisture contents of samples are listed in Table 1. Differences in the initial protein, fat, and moisture content of all the samples within a group were not significant (*p* > 0.05).

**Table 1:** Initial chemical compositions (protein, fat, and moisture) of the mixed pork patties. Data are presented as the mean ± *SD* of the mean (*n* = 3)

	Treatment			
	Control	BHT	VTE 0.1	VTE 0.3
Protein (%)	38.51 ± 2.44	36.58 ± 1.56	37.88 ± 2.12	36.50 ± 1.31
Fat (%)	5.39 ± 0.31	5.18 ± 0.25	5.14 ± 0.16	5.34 ± 0.22
Moisture (%)	39.22 ± 1.24	40.46 ± 2.51	39.39 ± 1.13	39.21 ± 1.58

### 3.4 pH analysis

As shown in Table 2, the pH values of all samples increased during refrigerated storage (*p* < 0.05), which may be due to the breakdown of proteins by microorganisms and enzymes (Ben Abdelmalek, Essid, Smeti, & Atti, 2018) and the nitrogenized compounds that were released by protein degradation (Zhang, Lin, Leng, Huang, & Zhou, 2013). The VTE‐treated groups had a lower pH than that of the control group (*p* < 0.05). This may be due to the antioxidant activity of VTE, which could inhibit the protein degradation.

**Table 2:** Effects of vine tea extract (VTE) on the pH of the mixed pork patties. Data are presented as the mean ± *SD* of the mean (*n* = 3)

	Storage time (day)	Treatment			
		Control	BHT	VTE 0.1	VTE 0.3
pH	0	6.47 ± 0.01^D^	6.47 ± 0.00^D^	6.47 ± 0.02^D^	6.47 ± 0.01^C^
	2	6.53 ± 0.02^Ca^	6.49 ± 0.01^CDab^	6.50 ± 0.01^CDab^	6.48 ± 0.02^BCb^
	4	6.55 ± 0.01^BCa^	6.51 ± 0.02^BCb^	6.52 ± 0.01^BCab^	6.49 ± 0.01^BCb^
	6	6.57 ± 0.00^ABa^	6.53 ± 0.00^ABb^	6.54 ± 0.00^ABb^	6.51 ± 0.01^ABc^
	8	6.59 ± 0.00^Aa^	6.55 ± 0.00^Ab^	6.56 ± 0.01^Ab^	6.53 ± 0.01^Ac^

^a‐d^Means within each row with different superscript letters are significantly different (*p * < 0.05).

^A‐D^Means within each column with different superscript letters are significantly different (*p*  < 0.05).

### 3.5 Color measurement

The L^*^ value of the mixed pork patties significantly decreased (*p* < 0.05) during refrigerated storage (Table 3), and the a^*^ and b^*^ values of the control and BHT groups significantly decreased (*p* < 0.05). Compared to the changes in the control group, the VTE‐treated samples maintained stable a^*^ and b^*^ values, even at lower a^*^ and b^*^ values (*p* < 0.05). This indicated that VTE had a protective effect on the color deterioration of the mixed pork patties during the storage period. Compared to those of the control group, the VTE‐treated samples showed significantly lower L^*^, a^*^, and b^*^ values (*p* < 0.05), and samples with higher added amounts of VTE had lower L^*^, a^*^, and b^*^ values. This may be due to the color (green) of the VTE.

**Table 3:** Effects of vine tea extract (VTE) on the color parameters of the mixed pork patties during the storage period. Data are presented as the mean ± *SD* of the mean (*n* = 3)

	Storage time (day)	Treatment			
		Control	BHT	VTE 0.1	VTE 0.3
L^*^	0	57.70 ± 0.04^Aa^	57.09 ± 0.06^Ab^	54.45 ± 0.23^Ac^	52.63 ± 0.11^Ad^
	2	56.15 ± 0.05^Ba^	55.14 ± 0.10^Bb^	51.67 ± 0.08^Bc^	52.24 ± 0.09^Ad^
	4	54.22 ± 0.13^Ca^	54.60 ± 0.31^Ca^	51.55 ± 0.12^BCb^	50.42 ± 0.15^Bc^
	6	53.23 ± 0.05^Da^	53.60 ± 0.21^Da^	51.17 ± 0.13^CDb^	49.65 ± 0.32^Cc^
	8	53.06 ± 0.12^Da^	53.27 ± 0.08^Da^	51.02 ± 0.16^Db^	47.92 ± 0.13^Dc^
a^*^	0	8.61 ± 0.07^Aa^	8.54 ± 0.06^Ab^	5.45 ± 0.32^c^	4.59 ± 0.06^d^
	2	8.14 ± 0.15^Bb^	8.46 ± 0.05^ABa^	5.53 ± 0.07^c^	4.64 ± 0.05^d^
	4	8.09 ± 0.06^Ba^	8.27 ± 0.04^Ba^	5.47 ± 0.04^b^	4.73 ± 0.31^c^
	6	7.22 ± 0.04^Cb^	7.85 ± 0.19^Ca^	5.30 ± 0.07^c^	4.71 ± 0.08^d^
	8	7.11 ± 0.03^Cb^	7.57 ± 0.03^Da^	5.39 ± 0.28^c^	4.58 ± 0.20^d^
b^*^	0	23.52 ± 0.05^Aa^	23.64 ± 0.07^Aa^	19.65 ± 0.36^b^	18.12 ± 0.06^c^
	2	23.07 ± 0.05^Bb^	23.44 ± 0.14^ABa^	19.60 ± 0.17^c^	18.01 ± 0.10^d^
	4	22.43 ± 0.14^Cb^	23.17 ± 0.09^BCa^	19.38 ± 0.03^c^	18.42 ± 0.29^d^
	6	22.18 ± 0.08^Cb^	22.94 ± 0.22^CDa^	19.43 ± 0.14^c^	18.27 ± 0.15^d^
	8	21.58 ± 0.18^Db^	22.63 ± 0.12^Da^	19.27 ± 0.36^c^	18.18 ± 0.27^d^

^a‐d^Means within each row with different superscript letters are significantly different (*p * <  0.05).

^A‐D^Means within each column with different superscript letters are significantly different (*p * <  0.05).

### 3.6 Effect of VTE on lipid oxidation of mixed pork patties

**Figure 3:**
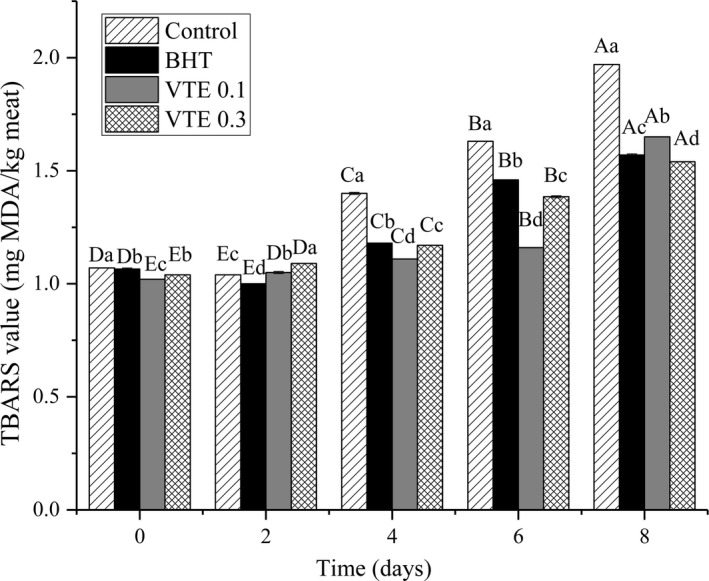
Effects of VTE on TBARS of the cooked mixed pork patties during refrigerated storage. ^a‐d^ Means within the same storage day between the different groups with different superscript letters are significantly different (*p * < 0. 05). ^A‐E^Means within the same group at different storage times with different superscript letters are significantly different (*p * <  0.05). All values are presented as the mean ± *SD* of three independent experiments

A change in the amount of TBARS can indirectly reflect the degree of oxidative rancidity in meat products (Turgut, Soyer, & Işıkçı, 2016). As shown in Figure 3, lipid oxidation occurred due to a significant increase (*p* < 0.05) in the TBARS value during refrigerated storage. The initial TBARS values of control, BHT, VTE 0.1, and VTE 0.3 groups were 1.07, 1.07, 1.02, and 1.04 mg MDA/kg meat, respectively. During refrigerated storage, compared to that of the control group, the other groups had lower TBARS values (*p* < 0.05), indicating that VTE could effectively inhibit the lipid oxidation of mixed pork patties. At the end of storage, the TBARS values of these groups were 1.97, 1.57, 1.65, and 1.54 mg MDA/kg meat, respectively. It was observed that VTE 0.3 group, with a higher added amount of VTE, had a higher inhibitory effect on lipid oxidation than did the BHT group (*p* < 0.05), and the ranking order of TBARS value was control > VTE 0.1 > BHT > VTE 0.3 in descending order. However, at 0–6 days of refrigeration, the TBARS value of VTE 0.1 group was lower than that of VTE 0.3 group (*p* < 0.05), while on day 8, the TBARS value of VTE 0.3 group was lower than that of VTE 0.1 group (*p* < 0.05). This phenomenon may be due to the fact that VTE with low concentration can rapidly diffuse into the samples to inhibit lipid oxidation, but its inhibition time is short; while VTE with high concentration releases slowly, it can sustainably inhibit the occurrence of lipid oxidation. The effective inhibition of VTE against lipid oxidation may be due to the presence of dihydromyricetin. Previous studies have reported that dihydromyricetin can effectively inhibit the oxidation of soybean oil (Ye et al., 2015).

### 3.7 Effects of VTE on protein oxidation of mixed pork patties

Protein oxidation can also cause meat deterioration (Lund, Heinonen, Baron, & Estévez, 2010). The changes of carbonyl and sulfhydryl contents can indirectly reflect the degree of protein oxidation in meat products.

**Figure 4:**
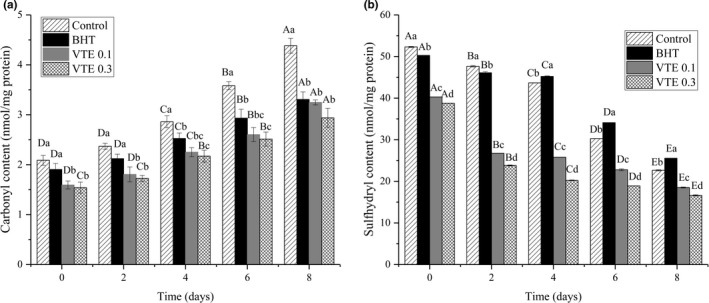
Effects of VTE on carbonyl content (a) and sulfhydryl content (b) of the cooked mixed pork patties during refrigerated storage. ^a‐d^Means within the same storage day between the different groups with different superscript letters are significantly different (*p*  <  0.05). ^A‐E^Means within the same group at different storage times with different superscript letters are significantly different (*p*  <  0.05). All values are presented as the mean ± *SD* of three independent experiments

As shown in Figure 4a, the protein carbonyl content increased significantly (*p* < 0.05) during refrigerated storage. The initial carbonyl contents of control, BHT, VTE 0.1, and VTE 0.3 groups were 2.09, 1.90, 1.59, and 1.54 nmol/mg protein, respectively. Compared with the control group, VTE showed strong inhibition on carbonyl content from the beginning of storage (*p* < 0.05). When refrigerated for 8 days, the final carbonyl contents of control, BHT, VTE 0.1, and VTE 0.3 groups were 4.38, 3.31, 3.25, and 2.94 nmol/mg protein, respectively. It was observed that the carbonyl content in the control group was significantly higher than that in the VTE treatment group. Obviously, 0.1% and 0.3% VTE significantly inhibited the formation of protein carbonyl compounds (*p* < 0.05) in the mixed pork patties over the entire storage period compared with that of the control group, and the inhibition was stronger than that of the BHT group (*p* < 0.05). In addition, protein oxidation occurred more rapidly than did lipid oxidation.

The decrease in the amount of sulfhydryl groups is used as another indicator of protein oxidation (He et al., 2018). The reduction of the sulfhydryl groups is due to the oxidation of the cysteine sulfhydryl group to form a disulfide bond, which induces protein cross‐linking (Jia, Kong, Liu, Diao, & Xia, 2012). As shown in Figure 4b, the sulfhydryl contents of all groups were significantly reduced (*p* < 0.05) during refrigerated storage. It could be observed that the sulfhydryl contents of the VTE‐treated samples were significantly lower than those of the control and BHT groups (*p* < 0.05) during refrigerated storage, and the rate of decline was faster in the VTE‐treated samples, which is similar to the findings of Jongberg, Skov, Tørngren, Skibsted, and Lund (2011). They found that adding white grape extract to chill stored beef patties also accelerated the sulfhydryl loss. In addition, Jongberg, Tørngren, Gunvig, Skibsted, and Lund (2013) found no protective effect of green tea extract against sulfhydryl loss in Bologna sausage. This may be due to the interactions of the sulfhydryl groups with the phenolic compounds. The ortho‐phenolic structure readily forms covalent bonds with nucleophilic sulfhydryl groups to form sulfhydryl‐oxime adducts, resulting in sulfhydryl loss. Studies have shown that phenolic substances exist in VTE (Ye et al., 2015), and we speculate that these phenolic substances may be combined with sulfhydryl groups, resulting in the lower sulfhydryl content in the VTE‐treated mixed pork patties than in the control group.

### 3.8 Texture profile analysis

Changes in sample hardness during refrigeration are shown in Table 4. The initial hardness of each group did not change significantly (*p* > 0.05) during refrigerated storage. However, the hardness of the control group significantly increased (*p* < 0.05), and the hardness of both the BHT group and VTE 0.3 group increased, but these changes were not significant (*p* > 0.05). The samples with a higher added mount of VTE had a lower hardness (*p* < 0.05), suggesting that VTE could inhibit the increasing hardness of the mixed pork patties during refrigerated storage. Previous studies showed that the separation of water and fat from the protein matrix caused emulsion instability, resulting in an increase in the hardness of meat products (Estévez, Ventanas, & Cava, 2005). Other studies suggested that protein oxidation affected protein function (Xiong, 2000). Furthermore, protein oxidation affected the protein solubility, resulting in aggregation and complex formation due to protein cross‐linking (Ganhão, Morcuende, & Estévez, 2010). The loss of protein function and the formation of cross‐linking between proteins led to an increase in the hardness of meat products. (Estévez et al., 2005). The results of this experiment showed that VTE had strong antioxidant activity, which could significantly inhibit protein oxidation. VTE may inhibit the increase in mixed pork patties hardness by protecting protein function and inhibiting protein cross‐linking. In addition, natural antioxidants can maintain the membrane integrity of muscle fibers and prevent water loss, thus maintaining the texture of meat products (Maqsood, Benjakul, & Balange, 2012). The results also showed that VTE could significantly inhibit lipid oxidation, so it is reasonable that VTE could inhibit the increasing hardness of the mixed pork patties. In addition, the springiness of all groups slightly decreased, but there were no significant differences (*p* > 0.05).

**Table 4:** Effects of vine tea extract (VTE) on the hardness and springiness of the mixed pork patties during the storage period. Data are presented as the mean ± *SD* of the mean (*n* = 3)

	Storage time (day)	Treatment			
		Control	BHT	VTE 0.1	VTE 0.3
Hardness (*N*)	0	374.37 ± 11.58^D^	370.23 ± 17.79	363.20 ± 13.23^B^	361.13 ± 16.00
	2	392.93 ± 8.31^CD^	378.53 ± 12.32	375.93 ± 9.12^B^	371.25 ± 12.54
	4	411.60 ± 12.09^BC^	393.17 ± 19.88	388.83 ± 12.45^AB^	383.96 ± 18.01
	6	433.70 ± 13.39^ABa^	405.83 ± 15.92^ab^	397.87 ± 14.22^ABb^	392.27 ± 14.40^b^
	8	459.00 ± 9.78^Aa^	409.27 ± 13.75^b^	416.63 ± 18.94^Aab^	400.43 ± 21.13^b^
Springines (cm)	0	4.42 ± 0.04	4.41 ± 0.09	4.43 ± 0.11	4.42 ± 0.06
	2	4.32 ± 0.05	4.34 ± 0.08	4.38 ± 0.10	4.36 ± 0.09
	4	4.27 ± 0.03	4.26 ± 0.06	4.27 ± 0.07	4.30 ± 0.11
	6	4.22 ± 0.14	4.21 ± 0.17	4.15 ± 0.13	4.23 ± 0.09
	8	4.19 ± 0.12	4.14 ± 0.10	4.13 ± 0.14	4.17 ± 0.11

^a‐d^Means within each row with different superscript letters are significantly different (*p*  <  0.05).

^A‐D^Means within each column with different superscript letters are significantly different (*p * <  0.05).

### 3.9 Microscopical observations

**Figure 5:**
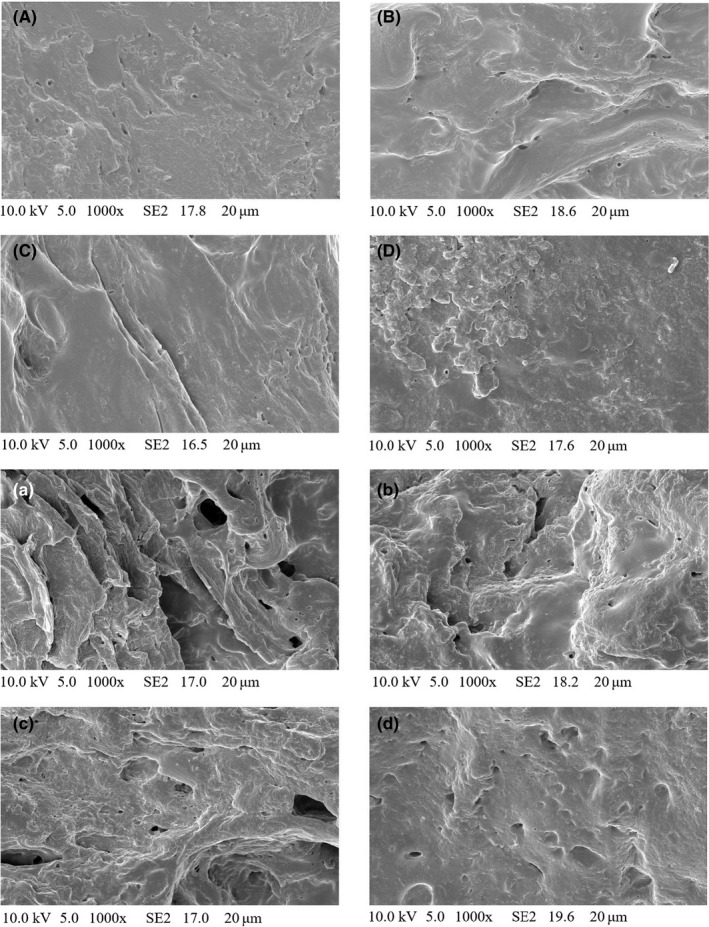
*SEM* images of all groups at 0 and 8 days of refrigeration. ([A–D] is the *SEM* of the control, BHT, VTE 0.1, and VTE 0.3 groups at 0 days of refrigeration; [a–d] is the *SEM* of the control, BHT, VTE 0.1, and VTE 0.3 groups at 8 days of refrigeration)

Figure 5 is the *SEM* images of all groups at 0 and 8 days of refrigeration. As shown in Figure 5, the initial *SEM* images of all the groups were smooth and flat; when refrigerated for 8 days, the *SEM* images of all the groups were rougher and uneven. This may be due to the oxidation of the mixed pork patties at 8 days of refrigeration, which destroyed their original structure. In addition, when refrigerated for 8 days, the VTE‐treated mixed pork patties were less damaged than that of the control group, suggesting that VTE was effective for suppressing the oxidation of the mixed pork patties during refrigerated storage.

### 3.10 Sensory evaluation

**Figure 6:**
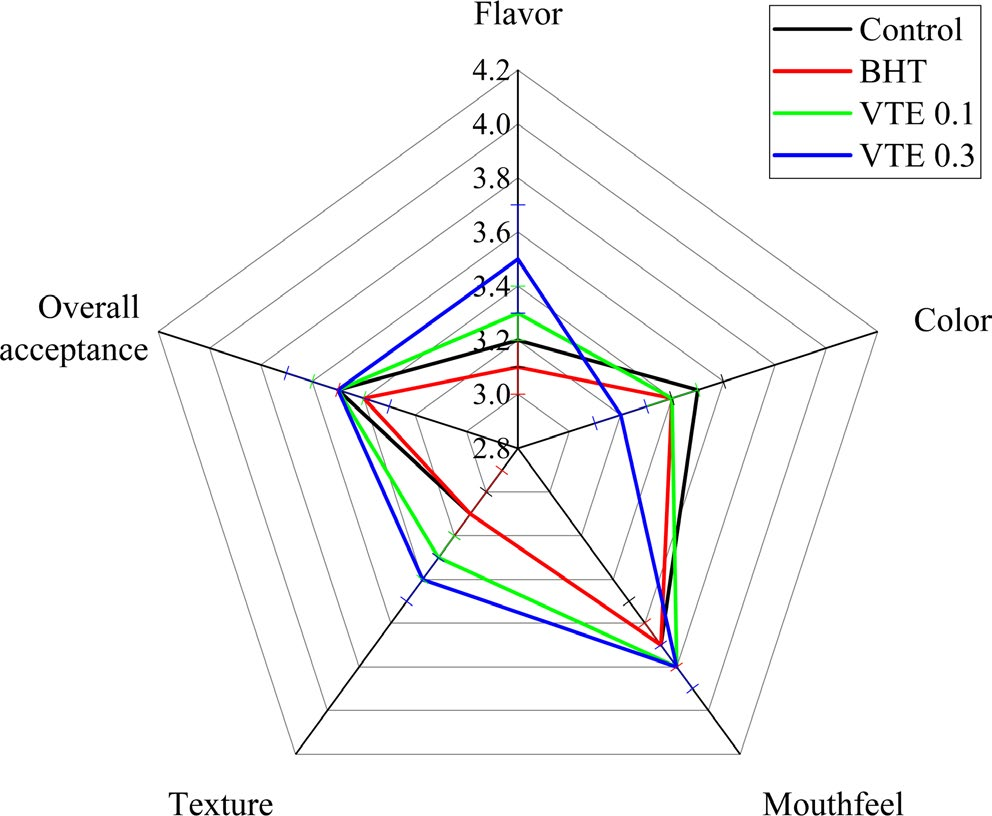
Effect of VTE on the sensory attributes of the cooked mixed pork patties. All values are presented as the mean ± *SD* of three independent experiments

The sensory characteristics of the mixed pork patties on day 0, including flavor, surface color, mouthfeel, texture, and overall acceptability, are shown in Figure 6. As the added amounts of VTE increased in the mixed pork patties, the flavor scores increased, which may be due to the inhibition of oxidation in the samples by VTE. However, the higher the added amounts of VTE, the lower the color scores were, possibly due to the color of the phytochemical itself. It was found that the mixed pork patties had a higher mouthfeel score, which may be due to the addition of soy protein meat analogues. The meat analogues are fibrous and have a good chewiness and meaty feeling, which resulted in the mixed pork patties having a good mouthfeel. The VTE‐treated samples had a high texture score, which is consistent with the above hardness results. In addition, overall acceptability was not affected by the addition of VTE. These results indicated that VTE can be added to mixed pork patties without adverse effects on the sensory attributes.

## 4 DISCUSSION

Previous studies have reported that natural antioxidants can significantly inhibit lipid and protein oxidation in meat products (Jiao, Zhao, Lin, & Wang, 2017; Rodríguez‐Carpena, Morcuende, & Estévez, 2011). The antioxidant activity of these natural antioxidants may be related to the presence of phenolic compounds. Flavonoids, a kind of phenolic antioxidant (Mattila, Hellström, Karhu, Pihlava, & Veteläinen, 2016), can scavenge free radicals (Jia et al., 2012). In this study, according to the results of GC‐MS, the main flavonoid of VTE was dihydromyricetin. This experiment found that VTE can effectively improve the quality of mixed pork patties, which may be due to the abundance of dihydromyricetin in VTE. Previous studies have reported that dihydromyricetin has a stronger antioxidant activity than that of BHA and TBHQ (Ye et al., 2015). As an antioxidant, VTE not only inhibited lipid and protein oxidation in the mixed pork patties, but also maintained the sensory properties and quality and represents a more effective utilization plant resources.

As a synthetic antioxidant, BHT is readily available and has been widely used to inhibit meat oxidation (Formanek et al., 2001). However, recent studies have shown that BHT is associated with health risks due to toxic effects (Lindenschmidt, Tryka, Goad, & Witschi, 1986). As a natural antioxidant, VTE not only has significant antioxidant activity (Gao et al., 2009), but can effectively improve meat quality without adverse effects. However, so far, no relevant literatures have reported the maximum permissible dosage of VTE. In this experiment, the dosage of VTE was much higher than that of BHT for the following two reasons: (a) International Food Standards stipulate that the maximum dosage of BHT in meat was 0.01% (Codex Alimentarius commission., 2015); therefore, we chose the maximum dosage of BHT 0.01% to evaluate the antioxidant activity of VTE; (b) In addition, the dosage of natural extracts in meat ranged from 0.1% to 1.5% in more than half of the literatures (Falowo, Fayemi, & Muchenje, 2014; Ribeiro et al., 2018), so we initially selected the dosages of VTE as 0.1%, 0.3%, and 0.5%. However, we found that when the dosage of VTE was 0.5%, the color of mixed pork patties was too dark, which affected the overall acceptability of the scoring group members. Therefore, we chose 0.1% and 0.3% as our experimental dosages.

Studies have shown that the physicochemical properties of natural antioxidants, such as solubility and stability, affect their antioxidant activity. In the experiment, we found that the solubility of VTE was poor. Meanwhile, the research showed that the effect of dissolving VTE with water‐containing ethanol (20%) was not ideal, which may affect the utilization of VTE (Ye et al., 2015). Currently, studies have shown that natural antioxidants have good thermal stability (Zhao et al., 2019), but the temperature range of VTE for thermal stability is uncertain. In addition, different cooking methods can also affect the effects of natural antioxidants (Zhao et al., 2019). Currently, the heat cooking mainly includes boiling, steaming, frying, baking, and microwave technology (Palermo et al., 2013). Studies showed that the loss of flavonols in foods increased during boiling, frying, and microwave ovens (Moreno, López‐Berenguer, & García‐Viguera, 2007; Zhao et al., 2019). Interestingly, different higher baking conditions did not lead to the degradation of flavonols (Yang et al., 2019). In this experiment, we used baking to cook mixed pork patties. We are not sure whether high temperature baking has an effect on the antioxidant activity of VTE. But as the main component of VTE, studies have shown that dihydromyricetin has good thermal stability, even better than TBHQ, and can be used as an antioxidant in edible oils and baked foods (Gao et al., 2009). Therefore, we speculate that high temperature has no significant effect on the thermal stability and antioxidant activity of VTE. Of course, this still needs further research to verify. Future studies on the physicochemical properties of VTE and the effects of different cooking methods on the effectiveness of VTE can further help to assess the potential application of VTE as an antioxidant in food.

## 5 CONCLUSIONS

This study showed that vine tea extract had a strong antioxidant activity, and it could effectively improve the quality characteristics of mixed pork patties and delay lipid oxidation and protein oxidation in the cooked mixed pork patties during refrigerated storage. The study provides a theoretical basis for extending the shelf life of meat products and developing healthy and safe antioxidants for the meat industry, which may play a role in promoting the development of meat industry.

## ETHICAL APPROVAL

This study does not involve any human or animal testing.

## CONFLICT OF INTEREST

All authors declare no conflicts of interest.

Informed consent: Written informed consent was obtained from all study participants.
